# Magnetic resonance imaging in women recently diagnosed with breast cancer. Where are we headed?

**DOI:** 10.1590/0100-3984.2019.52.4e1

**Published:** 2019

**Authors:** Fabiola Kestelman

**Affiliations:** 1 Radiologist at Clínica Cavallieri and at Clínica São Vicente da Gávea, Rio de Janeiro, RJ, Brazil. Email: fabkest@gmail.com.

In Brazil, an upper-middle-income country, the incidence of breast cancer has been increasing in the last decade, as it has in most low- and middle-income countries, reflecting an aging population, a change in reproductive habits, and lifestyle changes. The incidence of the disease is still considerably lower in Brazil than in most high-income countries^(^^1^^)^. However, the associated mortality is as high in Brazil as it is in many high-income countries, although it has been increasing in all age groups in Brazil since 1979, contrasting sharply with the decreases observed in most high-income countries since the early 1990s^(^^2^^)^.

The use of magnetic resonance imaging (MRI) in the context of breast cancer has gained prominence in recent years^(^^3^^-^^6^^)^. However, in patients with a recent diagnosis of breast cancer, certain indications regarding the evaluation of additional lesions in the affected breast, as well as in the contralateral breast, are controversial. When preoperative MRI of the breast was becoming increasingly popular among surgeons, two large studies were conducted evaluating the role of preoperative MRI in women with early-stage breast cancer^(^^7^^,^^8^^)^. In the comparative effectiveness of MRI in breast cancer (COMICE) study, conducted in the United Kingdom, 1623 women scheduled for surgery to treat primary breast cancer were randomized to undergo preoperative MRI or not^(^^7^^)^. The authors found no statistically significant difference between the two groups in terms of the main outcome measure, which was re-excision for positive margins, or in terms of disease-free survival at three years of follow-up. The MRI mammography of nonpalpable breast tumours (MONET) study, conducted in the Netherlands and involving 418 patients, evaluated whether preoperative MRI, in addition to mammography, ultrasonography, or both, reduced the number of surgical re-excisions^(^^8^^)^. Paradoxically, the addition of breast MRI was associated with an increased rate of re-excision for positive margins after breast-conserving surgery. The authors concluded that breast MRI should not be used in the preoperative routine for women with early-stage lesions.

The fact that neither the COMICE study nor the MONET study showed any benefit for preoperative breast MRI was unexpected. However, both studies had a number of methodological limitations. First, MRI was performed at a time when it was a relatively new technology. The level of expertise of the radiologists who interpreted the MRI examinations also varied widely from center to center. Second, the surgeons who enrolled patients in the COMICE study had no experience with preoperative MRI of the breast. Third, additional concerns were raised about unexpectedly low re-excision rates in the studies (approximately 19% in each group). Fourth, many of the participating centers did not have the capacity to perform MRI-guided biopsy (a necessary condition for the investigation of lesions identified only on MRI) before the selection of the surgical approach.

To compile the data and evaluate the various studies on the subject, Houssami et al.^(^^9^^)^ conducted a systematic meta- analysis of 19 studies involving a collective total of 2160 women of all ages with breast cancer, assessing the precision and surgical impact of preoperative breast MRI. In that study, the proportion of patients in whom the surgical approach was changed from breast-conserving surgery to mastectomy was 11.3% (95% CI: 6.8-18.3%). Additional tumor foci were found in the ipsilateral breast in 11-31% of the patients and in the contralateral breast in 3-6%. However, the authors observed no difference in the main outcome measure or improvement in disease-free survival. However, one critique of that meta-analysis is that it included only three randomized trials.

Before any definite conclusions can be drawn about the benefits of preoperative MRI, there is a need for studies that take into account the grade and aggressiveness of the disease; tumor biology and receptor status; tumor histology or morphology (especially invasive lobular carcinoma); breast density; family history; lymphovascular invasion; and multifocal or multicentric disease. Despite the limitations of preoperative MRI reported in the literature, there is evidence that the examination has clinical benefits in certain tumor subtypes. Studies of the use of preoperative MRI in women with triple-negative tumors have suggested that outcomes such as local recurrence and disease-free survival are poorer among women who do not undergo MRI prior to surgery^(^^10^^)^.

In the study conducted by França et al.^(^^11^^)^, published in this issue of Radiologia Brasileira, the use of preoperative MRI was evaluated in 61 patients. The MRI findings resulted in a change in practice in 23% of the patients, having a positive impact in 82.7% of those cases.

In conclusion, breast MRI is essential for surgical planning, assuming that it is employed correctly. The application of outdated guidelines to a new situation can result in unnecessary mastectomies. Guidelines that recommend mastectomy for multicentric breast cancer were established prior to the advent of MRI. It can be inappropriate to use the same guidelines for the management of multicentric foci of breast cancer detected by MRI, mainly because such foci, if small, can be adequately treated with radiotherapy. Randomized studies involving significantly larger samples and evaluating subpopulations, in order to determine which subgroups benefit most from the method, could provide novel conclusions.
